# A Fast Demodulation Algorithm for Fibre Bragg Grating Based on the TimeMixer-LightGBM Hybrid Learning Framework

**DOI:** 10.3390/s26134235

**Published:** 2026-07-03

**Authors:** Hang Gao, Yizhe Su, Kai Qian, Da Qiu, Song Liu, Tingting Zhang

**Affiliations:** School of Intelligent Systems Science and Engineering, Hubei Minzu University, Enshi 445000, China; topdredg@foxmail.com (H.G.); suyizhe2004@163.com (Y.S.); kaiqian@hbmzu.edu.cn (K.Q.); qiuda2008@163.com (D.Q.); 2002016@hbmzu.edu.cn (S.L.)

**Keywords:** fibre Bragg grating (FBG) demodulation, TimeMixer, LightGBM, hybrid learning framework

## Abstract

Fibre Bragg Grating (FBG) demodulation technology is central to structural health monitoring. However, spectral distortion and noise caused by complex environments, along with the challenge of balancing accuracy and real-time performance in existing deep learning algorithms, severely limit its application in large-scale dynamic sensing networks. To address this challenge, this paper proposes a hybrid learning framework named TimeMixer-LightGBM. The framework first employs a pure MLP-based TimeMixer model to efficiently extract multi-scale spectral sequence features from FBG reflection spectra, and then uses a LightGBM model (gradient boosted decision trees) to perform fast regression on these features for centre wavelength shift prediction. Experiments on synthetically distorted FBG spectra (including asymmetric shape variations and additive white noise) show that the method achieves picometre-level accuracy (RMSE = 2.128 pm) with an average processing time of only 0.08 ms per spectrum, representing a speedup of about 4.5 times over the latest deep learning models for FBG demodulation. It also exhibits excellent noise robustness, maintaining an average absolute error of 1.5 pm. Ablation experiments confirm the necessity and synergy of the hybrid architecture. The framework was further applied to demodulate double-peaked overlapping spectra, outperforming existing methods under mild, moderate and severe overlap conditions while keeping inference times in the sub-millisecond range. This study provides a novel and effective technical solution for real-time high-precision FBG demodulation, validates the effectiveness of pure MLP architectures in spectral analysis, and lays a theoretical foundation for deploying such demodulation on embedded edge devices, thereby achieving a favourable balance of accuracy, speed and scalability.

## 1. Introduction

The demodulation principle of fibre Bragg gratings (FBGs) is centred on accurately measuring the shift of the Bragg wavelength, because this shift directly corresponds to the strain and temperature variations imposed on the FBGs. FBG demodulation technologies are widely applied in numerous monitoring fields owing to their high precision, immunity to electromagnetic interference, intrinsic safety, and ease of multiplexing and networking [1,2,3,4]. However, long-term operation of FBGs under environmental factors such as radiation and high temperature tends to cause spectral distortion and elevated noise, thereby affecting spectral stability and resulting in reflected spectrum distortion [5]. Moreover, light source fluctuations and interference noise in the demodulation system further degrade the signal quality [6]. Consequently, achieving high-precision demodulation of distorted spectra is critical to ensuring the reliability and stability of grating sensor networks.

Existing optimisation techniques for FBG reflected spectrum distortion can be divided into hardware and software levels. Hardware optimisation is mainly achieved by improving fabrication processes, grating structure design, and optimised packaging and installation [7], whereas software optimisation focuses on enhancing signal processing and demodulation algorithms [8]. Nevertheless, when FBGs are already deployed in complex environments (e.g., embedded in concrete structures, implanted in the human body, or sent deep into oil wells) or when inherent defects exist in the packaging process, physically modifying the sensors is often not feasible. In contrast, signal processing algorithms can directly postprocess the acquired distorted spectra without the need to replace sensors or interrupt the system, offering the significant advantages of low cost and rapid implementation.

By mining the statistical regularities behind data, machine learning can yield reliable and repeatable judgments. Traditional machine learning techniques, such as random forest regression [9], the sparrow search algorithm [10], and Gaussian mixture models [11], have been applied to FBG demodulation. With the rapid development of artificial intelligence, deep learning, in particular, has attracted widespread attention in the field of optical fibre sensing demodulation due to its powerful nonlinear feature extraction capability. Deep learning frameworks generally outperform traditional machine learning methods by adjusting the number of network layers and neurons, enabling them to more profoundly characterise the complex nonlinear relationships between inputs and outputs. Hao Jiang et al. [12] proposed an effective wavelength detection method based on a long short-term memory (LSTM) network, capable of achieving high-accuracy and high-speed wavelength detection under overlapping spectra. Xiangxin Shao et al. [13] proposed a novel method using a long short-term memory convolutional neural network (LSTM-CNN) to demodulate grating sensor spectra for the identification and monitoring of fire hazards in underground tunnels. Rui Tang et al. [14] proposed a demodulation method combining cubic spline interpolation (CSI) and a one-dimensional convolutional neural network (1-DCNN) model, which effectively identified distorted spectra using the 1-DCNN. Xianyu Lai et al. [15] proposed a deep learning method based on a CNN-LSTM architecture, specifically designed to address the challenges of high-precision temperature and pressure demodulation in side-hole fibre Bragg gratings (SHFBGs). Wenjuan Sheng et al. [16] employed a temporal convolutional network (TCN), a convolutional neural network (CNN), and a gated recurrent unit (GRU) to evaluate the strain of FBG sensors at the middle, positive electrode, and negative electrode of a battery, so as to improve the estimation accuracy of the battery’s state of charge. Wenjuan Sheng et al. [17] used a temporal convolutional network (TCN) to extract hidden information and long-term temporal relationships from input features, and then applied a Light Gradient Boosting Machine (LightGBM) for fast prediction of demodulation errors.

Although the aforementioned deep learning models have demonstrated remarkable accuracy advantages in the demodulation of distorted FBG spectra, these advantages often come at the cost of complex network structures and huge computational overhead. For instance, the convolution operations and multi-layer stacking of CNNs [15], as well as the sequential dependencies and complex gating mechanisms of LSTMs/GRUs [16], significantly increase computational latency. Even the TCN [17], which introduces parallel computation, often requires stacking multiple layers of dilated convolutions to guarantee a sufficiently large receptive field covering the full-spectrum features; this not only increases the number of parameters but also limits the achievement of ultimate inference speed. In time-sensitive application scenarios such as seismic monitoring [18], high-speed rotating machinery condition monitoring [19], and real-time load analysis of aerospace vehicles [20], traditional deep learning frameworks struggle to meet the sub-millisecond response requirements, and the bottleneck in demodulation speed severely constrains the practicality of the system.

To overcome the dual constraints of real-time performance and accuracy, this paper proposes a TimeMixer-LightGBM hybrid demodulation framework. The TimeMixer model adopts a pure multi-layer perceptron (MLP) architecture, whose core operations are highly parallel matrix multiplications that are more computationally efficient at the hardware level than convolution operations in traditional CNNs, laying a hardware foundation for inference speed improvement. Through its unique multi-scale mixing mechanism, TimeMixer efficiently decouples and extracts global profile information and local fine-grained features from signals [21]. While this architecture has been widely applied in time series forecasting [22,23], this study innovatively introduces it into spectral analysis, treating the FBG reflected spectrum as one-dimensional sequence data and using TimeMixer to extract multi-scale spectral features. Subsequently, LightGBM [24] performs rapid regression of the central wavelength, utilising gradient boosting trees with histogram-based acceleration and leaf-wise growth for ultra-high-speed prediction.

The key contributions are threefold: (i) a decoupled architecture combining TimeMixer and LightGBM that breaks the conventional end-to-end paradigm; (ii) a multi-scale bidirectional mixing mechanism that explicitly separates coarse-scale baseline drift from fine-scale asymmetric distortion, hierarchically decoupling overlapped peaks; and (iii) a pure MLP backbone combined with a lightweight tree-based regressor that is inherently compatible with embedded hardware such as FPGAs and DSPs. Single-peak demodulation experiments show that the framework achieves picometre-level accuracy with a processing time of 0.08 ms per spectrum (approximately 4.5 times faster than GAF-CNN-LSTM), while double-peak overlapping tests confirm its robust decoupling capability. Experimental results, detailed in Section 3, demonstrate that the framework simultaneously achieves picometre-level accuracy, microsecond-level latency, strong robustness to distortions and noise, and high suitability for edge deployment.

## 2. Materials and Methods

### 2.1. TimeMixer Model

**Figure 1:**
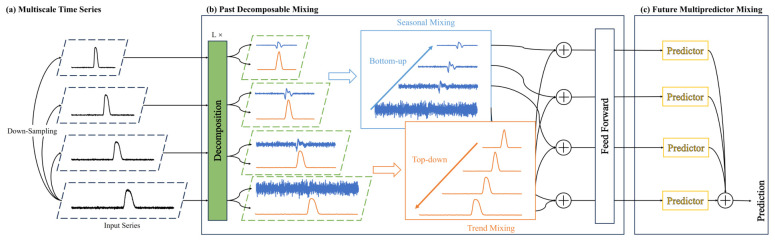
TimeMixer’s model architecture.

FBG reflection spectra naturally possess a one-dimensional sequential structure: the wavelength axis acts analogously to the time axis, and the reflectivity values form a sequence where neighbouring points are strongly correlated. Moreover, FBG signals exhibit an intrinsic multi-resolution nature. The global baseline and slow drift (e.g., due to temperature changes or bending) correspond to coarse-scale components, while the sharp peak, local asymmetries and fine distortions (e.g., caused by non-uniform strain fields) reside in fine-scale components. Therefore, a model that can simultaneously capture and decouple these two scales is highly desirable. TimeMixer [21] is precisely such a model: it is a sequence prediction architecture purely based on a multi-layer perceptron (MLP). Through its innovative multi-scale decomposition and mixing mechanism, it can explicitly separate the coarse-scale (trend) and fine-scale (seasonal) components, and then mix them bidirectionally (bottom-up for fine details, top-down for global trends). This design aligns exactly with the physical characteristics of FBG signals: the coarse scale captures the spectral envelope and baseline trend (robust against high-frequency noise), while the fine scale preserves the critical peak shape and subtle distortion features that determine the exact Bragg wavelength. Moreover, unlike CNNs, which require designing kernel sizes and stacking many convolutional layers to enlarge the receptive field, and unlike RNNs/LSTMs, which suffer from inherent sequential dependencies that prevent parallel computation along the time dimension, the pure MLP architecture of TimeMixer has no such restrictions. Its core operations are matrix multiplications and fully-connected layers—both highly parallelisable on modern hardware (GPUs, TPUs, and even FPGAs). This hardware-friendly characteristic eliminates the computational bottlenecks of serial recurrence (LSTM) and the parameter redundancy of deep convolution stacks (CNNs), laying a solid theoretical foundation for achieving ultra-low inference latency. Figure 1 illustrates the model architecture of TimeMixer. The model first performs multi-scale decomposition on the input spectral sequence; subsequently, these multi-scale sequences are projected into deep features via embedding techniques; then, two major modules—Past-Decomposable-Mixing (PDM) and Future-Multipredictor-Mixing (FMM)—realise the multi-scale mixing prediction.

#### 2.1.1. Multi-Scale Decomposition

To obtain multi-scale spectral sequences, TimeMixer employs average pooling to downsample the input signal to $x\in R^{P\times C}$ scales, yielding a set of multi-scale sequences, as shown in Equation (1):

(1) $$X=\{x_{0},\cdots ,x_{M}\}$$

where $x_{m}\in R^{[\frac{P}{2^{m}}]\times C},m\in \left\{0,\cdots ,M\right\}$, *C* denotes the number of variables, $x_{0}=x$ is the input sequence containing the finest spectral details, and $x_{M}$ reflects the changes in the macroscopic profile of the spectrum. The downsampling operation for the *m*-th scale can be expressed as Equation (2):

(2) $$x_{m}\left[i,:\right]=\frac{1}{2}\left(x_{m-1}\left[2i-1,:\right]+x_{m-1}\left[2i,:\right]\right),\;i=1,\ldots ,\left\lfloor \frac{P}{2^{m}}\right\rfloor$$

where $x_{m-1}$ is the sequence from the previous scale. This operation progressively reduces the sequence length while preserving the overall shape. Then, through an embedding layer, these multi-scale sequences are projected into deep features $X^{0}$, expressed as $X^{0}=Embed\left(X\right)$. By means of the above design, we obtain a multi-scale representation of the input sequence.

#### 2.1.2. Past-Decomposable-Mixing (PDM) Module

Next, the past spectral information at different scales (corresponding to the “past” information in a time series model) is mixed through stacked PDM blocks. For the *l*-th layer, the input is $X^{l-1}$, and the PDM process can be formalised as Equation (3):

(3) $$X^{l}=PDM\left(X^{l-1}\right),\;l\in \left\{0,\cdots ,L\right\}$$

where *L* is the total number of layers, and $X^{l}=\left\{x_{0}^{l},\cdots ,x_{M}^{l}\right\},\;x_{m}^{l}\in R^{\left[\frac{P}{2^{m}}\right]\times d_{\mathrm{model}}}$ denotes the mixed past feature representation.

The PDM module decomposes the multi-scale time series $X^{l}$ into a seasonal part $S^{l}=\left\{s_{0}^{l},\cdots ,s_{M}^{l}\right\}$ and a trend part $T^{l}=\left\{t_{0}^{l},\cdots ,t_{M}^{l}\right\}$ through series decomposition. This decomposition can be performed via a moving average kernel: let Avg(x,k) denote the moving average of *x* with kernel size *k*. Then the trend component is extracted as Equation (4)

(4) $$t_{m}^{l}=\mathrm{Avg}\left(x_{m}^{l},k_{m}\right)$$

and the seasonal component is the residual:

(5) $$s_{m}^{l}=x_{m}^{l}-t_{m}^{l}$$

where $k_{m}$ is chosen proportional to the sequence length at scale *m* to ensure proper trend extraction. In the context of spectral analysis, the trend component $T^{l}$ corresponds to the baseline and overall profile of the spectrum, whereas the seasonal component $S^{l}$ captures high-frequency local details such as sidelobes and noise. Considering the distinct nature of the seasonal and trend components, mixing operations are applied to each of them separately to integrate multi-scale information.

**Figure 2:**
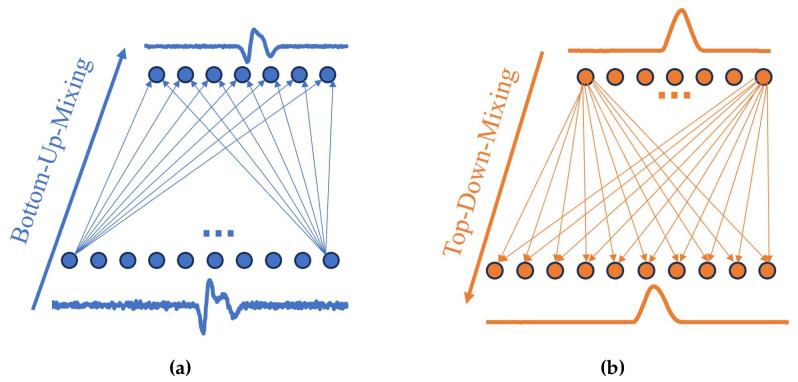
The temporal linear layers in seasonal and trend mixing: (**a**) The temporal linear layer in seasonal mixing. (**b**) The temporal linear layer in trend mixing.

In the seasonal mixing, a Bottom-Up approach is adopted, as shown in Figure 2a, which integrates fine-scale sequence information from lower levels upward, supplementing detailed information for coarser-scale seasonal modelling. The Bottom-Up mixing for the *m*-th scale can be formally expressed as Equation (6):

(6) $$s_{m}^{l}=s_{m}^{l}+F_{\mathrm{bu}}\left(s_{m-1}^{l}\right)$$

where $F_{\mathrm{bu}}\left(\cdot \right)$ employs a two-layer linear layer along the time dimension with a GELU activation function in between. Explicitly, for a given input *u*, the operation is

(7) $$F_{\mathrm{bu}}\left(u\right)=W_{2}^{\mathrm{bu}}\cdot \mathrm{GELU}\left(W_{1}^{\mathrm{bu}}\cdot u+b_{1}^{\mathrm{bu}}\right)+b_{2}^{\mathrm{bu}}$$

This bottom-up propagation injects fine-scale peak details (e.g., sharpness and asymmetry) into coarser scales, which is essential for preserving subtle distortion features that would otherwise be smoothed out by downsampling.

In the trend mixing, a Top-Down approach is adopted, as shown in Figure 2b, which leverages macroscopic knowledge from coarser-grained data to guide the trend modelling of finer-grained data. The Top-Down mixing for the *m*-th scale can be formally expressed as Equation (8):

(8) $$t_{m}^{l}=t_{m}^{l}+F_{\mathrm{td}}\left(t_{m+1}^{l}\right)$$

where $F_{\mathrm{td}}\left(\cdot \right)$ also uses a two-layer linear layer with GELU activation. Its explicit form is analogous to $F_{\mathrm{bu}}$ but with its own parameters.

Finally, a residual connection and layer normalisation are applied within each PDM block to facilitate training stability:

(9) $$x_{m}^{l,\mathrm{out}}=\mathrm{LayerNorm}\left(x_{m}^{l}+\mathrm{Dropout}\left(s_{m}^{l}+t_{m}^{l}\right)\right)$$

where $x_{m}^{l,\mathrm{out}}$ is the output of the PDM block for scale *m*.

#### 2.1.3. Future-Multipredictor-Mixing (FMM) Module

After *L* PDM blocks, we obtain the multi-scale past information ${𝒳}^{L}=\left\{x_{0}^{L},\cdots ,x_{M}^{L}\right\}$, $x_{m}^{L}\in R^{\left\lfloor P/2^{m}\right\rfloor \times d_{\mathrm{model}}}$. Then, the multi-scale sequences are aggregated for prediction through the FMM block. The FMM process is as follows:

First, for multi-scale prediction, an independent predictor $P_{m}$ is used for each scale *m* to make predictions from the past information $x_{m}^{L}$, which is formally expressed as Equation (10):

(10) $${\hat{x}}_{m}=P_{m}\left(x_{m}^{L}\right),\;m\in \left\{0,\cdots ,M\right\}$$

where ${\hat{x}}_{m}\in R^{F\times C}$ represents the subsequent spectral prediction generated from the *m*-th scale sequence. In our framework, we do not directly use ${\hat{x}}_{m}$; instead, we extract the intermediate feature ${\hat{f}}_{m}$ before the final projection layer (see Section 2.3.2).

Next, the predictions are ensembled: all scale predictions are combined through a learnable weighted summation to yield the final prediction result:

(11) $$\hat{y}=\sum_{m=0}^{M}w_{m}\cdot {\hat{x}}_{m},\;\mathrm{where}\;w_{m}=\frac{\exp(\alpha_{m})}{\sum_{j=0}^{M}\exp\left(\alpha_{j}\right)}.$$

Here, $\alpha_{m}$ are learnable scalar parameters, and $w_{m}$ are the normalised weights.

### 2.2. LightGBM Model

**Figure 3:**
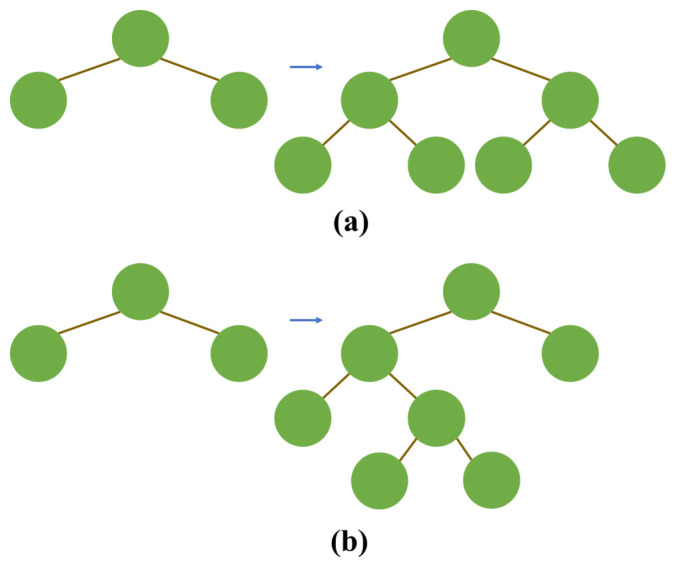
Decision tree growth strategies: (**a**) leaf-wise, (**b**) level-wise.

LightGBM (Light Gradient Boosting Machine) [24] is an efficient gradient boosting framework. Rather than detailing the general gradient boosting principle, we focus on its optimisations that make it particularly suitable for handling the high-dimensional features produced by TimeMixer. TimeMixer outputs a deep feature vector of dimension $d_{model}$ (e.g., 256) for each spectrum. While this representation is compact compared to the raw spectrum, it can still be moderately high-dimensional and may contain sparse or redundant components. LightGBM addresses these challenges through four key techniques:

- Histogram-Based Algorithm: Instead of scanning all data points to find the best split, LightGBM discretises continuous feature values into a fixed number of bins. This reduces the time complexity from O(#features×#data) to O(#features×#bins), which is crucial for fast training and inference on high-dimensional features.
- Gradient-Based One-Side Sampling (GOSS): GOSS retains samples with large gradients (i.e., those that are poorly predicted) and randomly samples small-gradient samples with weight compensation. This focuses the model’s capacity on the most informative features and samples, accelerating convergence without sacrificing accuracy.
- Exclusive Feature Bundling (EFB): EFB bundles mutually exclusive features (rarely non-zero simultaneously) into a single feature, reducing the effective dimensionality from #features to #bundles. This is particularly beneficial when TimeMixer produces sparse or redundant activations, as it compresses the feature space losslessly while lowering memory and computation.
- Leaf-Wise Leaf Growth Strategy: Unlike level-wise growth that splits all leaves at the same depth (Figure 3a), LightGBM adopts a leaf-wise growth strategy with depth constraint (Figure 3b). It selects the leaf with the largest split gain each iteration, achieving faster loss reduction. A maximum depth limit prevents overfitting.

### 2.3. TimeMixer-LightGBM Hybrid Learning Model Framework

**Figure 4:**
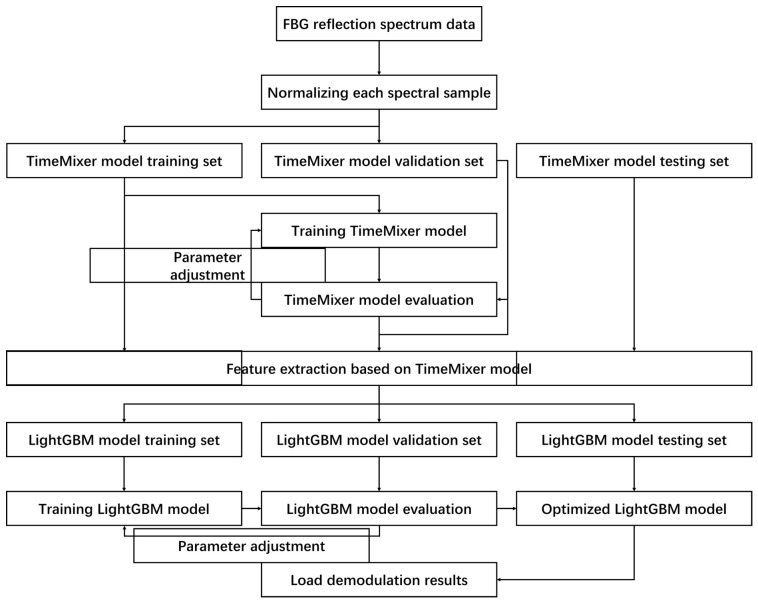
The overall flow of the TimeMixer-LightGBM model.

The TimeMixer-LightGBM hybrid demodulation framework proposed in this paper aims to address the core challenge in FBG sensing systems of simultaneously achieving high precision and low latency. To this end, the framework decouples the demodulation process into two stages: expressive feature extraction followed by flexible nonlinear regression. TimeMixer first transforms the raw spectrum into a structured, high-dimensional feature vector that captures multi-scale spectral characteristics (e.g., baseline trend, peak shape, and asymmetries). This representation is naturally suited for tree-based models like LightGBM, which excel at handling heterogeneous, high-dimensional inputs through threshold splitting. By separating the task into “feature extraction” (TimeMixer) and “regression” (LightGBM), our architecture leverages the strengths of both paradigms without sacrificing speed. Figure 4 illustrates the overall workflow based on the TimeMixer-LightGBM model, which consists of three core steps: dataset construction and preprocessing, deep feature extraction based on TimeMixer, and central wavelength regression based on LightGBM.

#### 2.3.1. Dataset Construction and Preprocessing

While the proposed algorithm is ultimately intended for real FBG systems, we employ a physics-based synthetic dataset as a controlled benchmark for algorithmic development. This approach is consistent with common practice in the field and allows systematic evaluation across a wide range of distortion parameters that are difficult to obtain from field measurements alone. The generalisation of the framework to real-world applications is discussed in Section 2.4.3.

To obtain a sufficient amount of FBG reflected spectral data that closely matches actual operating conditions, this study, with reference to [25], introduces an improved model based on the asymmetric generalised Gaussian function for spectral simulation. This model can flexibly characterise both normal and distorted spectra, and its mathematical expression is given by Equation (12):

(12) $$ℜ\left(\lambda \right)=\left\{\begin{array}{c} R_{0}\;\exp\left[-\left(2^{\nu }\ln2\right){\left|\frac{\lambda -\lambda_{B}}{W}\right|}^{\nu }\right],\;\lambda <\lambda_{B}\\ R_{0}\;\exp\left[-\left(2^{\nu }\ln2\right){\left|\frac{\lambda -\lambda_{B}}{\chi \cdot W}\right|}^{\nu }\right],\;\lambda \geq \lambda_{B} \end{array}\right.$$

where λ represents the wavelength variable, $R_{0}$ is the peak reflectivity at the Bragg wavelength $\lambda_{B}$, and W is the full width at half maximum (FWHM) of the spectrum, measured in nanometres (nm). The shape factor ν controls the peak sharpness (ν=2 gives a Gaussian shape, ν>2 produces a flattened “super-Gaussian” peak, and ν<2 yields a sharper peak), while the asymmetry factor χ adjusts the left/right bandwidth ratio (χ=1 for symmetric, χ>1 for left-steep/right-tailed, χ<1 for the opposite). Equation (12) models the main lobe of the FBG reflection spectrum and does not include sidelobes. This simplification is sufficient for demodulation algorithm development, where the centre wavelength is determined by the main peak position. During the data generation process, parameters are randomly selected within defined ranges, and random Gaussian white noise is added to simulate the actual measurement environment. All generated spectral data undergo standardised preprocessing and are divided into training, validation, and test sets in proportion to ensure the effectiveness of model training and the reliability of evaluation.

**Figure 5:**
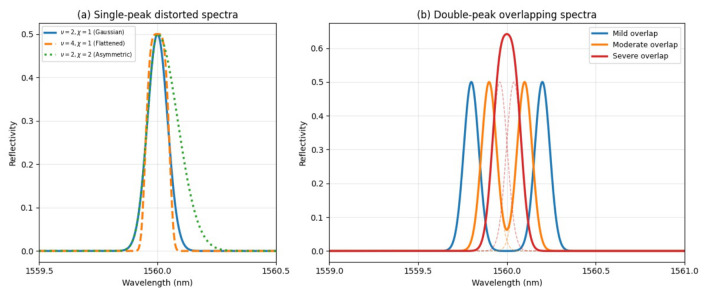
Representative synthetic FBG spectra generated by Equation (12). (**a**) Single-peak spectra with different shape factor ν and asymmetry factor χ values. (**b**) Double-peak overlapping spectra for mild-, moderate-, and severe-overlap conditions, with centre wavelength spacings δλ of 0.40 nm, 0.20 nm, and 0.08 nm, respectively.

To illustrate the spectral variability captured by this model, Figure 5 shows representative examples of single-peak and double-peak overlapping spectra generated from Equation (12). In panel (a), the blue solid line represents the standard Gaussian profile (ν=2,χ=1), serving as a reference; the yellow dashed line shows a flattened peak (ν=4,χ=1); and the green dotted line illustrates an asymmetric spectrum with right-side broadening (ν=2,χ=2). In panel (b), three overlap levels—mild (δλ=0.40 nm), moderate (δλ=0.20 nm), and severe (δλ=0.08 nm)—are shown, where the dashed lines indicate the individual peaks and the solid lines represent the combined spectra. These examples confirm that the adopted model captures a wide range of physically meaningful spectral distortions and overlap conditions, providing a reliable foundation for the subsequent algorithm evaluation.

#### 2.3.2. Feature Extraction with TimeMixer and Wavelength Prediction via LightGBM

This study innovatively employs the TimeMixer model as a deep feature extractor. The multi-scale decomposition and mixing mechanism of TimeMixer is highly suitable for processing sequence signals such as FBG reflected spectra, which contain complex patterns.

In the first stage, the TimeMixer model is trained on the training set. During this stage, the task of TimeMixer is spectral sequence prediction/reconstruction, i.e., learning the intrinsic correlations among the wavelength points of the spectrum. The validation set is used to evaluate model performance and to select the optimal model architecture and hyperparameters.

In the second stage, after its generalisation capability is verified on the test set, the parameters of the well-trained TimeMixer model are frozen and used as a fixed feature extractor, which is then applied to the entire dataset to batch-obtain the corresponding deep features.

The core operation of feature extraction resides in the FMM module of TimeMixer. Within this module, the independent predictor at each scale consists of two parts: a prediction layer and a projection layer. For the predictor at the *m*-th scale, its prediction layer is responsible for regressing the future sequence ${\hat{f}}_{m}$ from the past information $x_{m}^{L}$ at that scale, a process that can be formally expressed as Equation (13):

(13) $${\hat{f}}_{m}=\mathrm{Predict}\left(x_{m}^{L}\right),\;m\in \left\{0,\cdots ,M\right\}$$

Subsequently, the projection layer projects the $d_{\mathrm{model}}$ deep representations output by the prediction layer to *C* target variable dimensions, generating the final prediction ${\hat{x}}_{m}$ for that scale, as formalised in Equation (14):

(14) $${\hat{x}}_{m}=\mathrm{Projection}\left({\hat{f}}_{m}\right),\;m\in \left\{0,\cdots ,M\right\}$$

**Figure 6:**
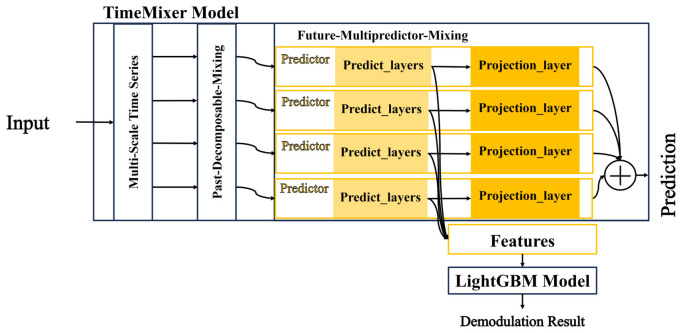
TimeMixer’s feature extraction and LightGBM’s wavelength prediction framework.

Figure 6 illustrates the feature extraction with TimeMixer and wavelength prediction with LightGBM framework. This study extracts the output of the prediction layer, ${\hat{f}}_{m}$, from each predictor as the deep feature refined from the spectral sequence at that scale. The ${\hat{f}}_{m}$ outputs from all scales are concatenated to collectively constitute the feature input $F^{L}=\left\{f_{0}^{l},\cdots ,f_{M}^{l}\right\}$ for the LightGBM model. Consequently, the features extracted from the TimeMixer training set form the new training set for LightGBM, while the features extracted from the TimeMixer validation and test sets are correspondingly transformed into the validation and test sets for LightGBM.

Finally, the newly constructed feature dataset is fed into the LightGBM model for central wavelength prediction. Leveraging optimisation techniques such as its histogram-based algorithm, LightGBM can efficiently process the high-dimensional features generated by TimeMixer, enabling fast and accurate regression of the wavelength shift.

### 2.4. Experimental Environment and Dataset Construction

The experimental environment of this study was based on a high-performance computing platform equipped with an AMD Ryzen 5 7500F 6-Core Processor @3.70 GHz, 32 GB of memory, and an NVIDIA GeForce RTX 4060 Ti GPU (8 GB VRAM). The software environment utilised the Windows 11 operating system, with deep learning frameworks built on Anaconda 24.11.3, PyTorch 2.4.4, and GPU acceleration via CUDA 12.6. The tree model was implemented using LightGBM 4.6.0. All code was written in Python 3.8.20.

#### 2.4.1. Single-Peak Distorted Spectrum Dataset

To construct a reliable training and testing benchmark, 10,000 FBG reflected spectral samples were generated using the improved model based on the asymmetric generalised Gaussian function (Equation (12)). The model parameters are physically meaningful for FBG spectral distortions. The shape factors ν were randomly selected from [2, 4] (covering ideal Gaussian to severely flattened peaks), and the asymmetry factor χ was randomly chosen from [1, 3] (covering typical asymmetry levels). The central wavelength was randomly set within [1559, 1561] nm. Each sample contained 2001 data points with a step size of 0.001 nm. The full width at half maximum (FWHM) was fixed at 0.2 nm. Peak reflectivity $R_{0}$ was randomly chosen between 0.5 and 1.0. Additive Gaussian white noise was added with SNR randomly selected in the range of 20–35 dB. The dataset was divided into training, validation, and test sets in a 70%/15%/15% ratio. All spectra were per-sample Z-score-normalised before model training.

The parameter ranges were determined based on both the literature and practical considerations. The shape factor ν∈[2,4] was chosen because ν=2 corresponds to the ideal Gaussian profile commonly assumed for apodised FBGs, while ν>2 produces a flattened super-Gaussian shape that has been observed in FBG spectra under non-uniform strain fields or grating saturation. The asymmetry factor χ∈[1,3] was selected to cover the typical skewness levels reported in practical FBG sensors under thermal or mechanical gradients. The SNR range of 20–35 dB reflects typical signal quality levels in industrial FBG interrogator deployments, where 20 dB represents a moderately noisy field environment and 35 dB corresponds to a well-controlled laboratory setting. All spectra were per-sample Z-score-normalised before model training.

#### 2.4.2. Double-Peak Overlapping Spectrum Dataset

A double-peak overlapping spectrum dataset was further constructed on the basis of the single-peak data. Double-peak spectra were formed by the linear superposition of two independently generated FBG reflected spectra, with each single peak still adopting the asymmetric generalised Gaussian model. The spectral acquisition parameters were entirely consistent with the single-peak dataset: each sample contained 2001 data points covering 1559–1561 nm with a step of 0.001 nm. The physical parameters of a single FBG peak are: shape factor ν∈[2,4], asymmetry factor χ∈[1,2], peak reflectivity fixed at 0.5, and half-width fixed at 0.1 nm. SNR was randomly selected in the range of 5–40 dB. Each double-peak sample contained two Bragg wavelength labels $\left(\lambda_{B1},\lambda_{B2}\right)$ ($\lambda_{B1}\leq \lambda_{B2}$). Based on the spacing $\Delta \lambda =|\lambda_{B2}-\lambda_{B1}|$, three overlapping levels were defined:

- Mild overlap: Δλ∈[0.30,0.50] nm.
- Moderate overlap: Δλ∈[0.15,0.30] nm.
- Severe overlap: Δλ∈[0.02,0.15] nm.

For each level, 10,000 samples were independently generated and split into training/validation/test sets (7000/1500/1500). All data were per-sample Z-score-standardised. The double-peak spectra were formed by the linear superposition of two independently generated FBG reflected spectra, with each single peak adopting the asymmetric generalised Gaussian model described in Section 2.3.1. This linear superposition approach has been widely adopted in FBG demodulation algorithm development. In a recent study, Lai et al. [15] employed identical linear superposition of two Gaussian functions to generate overlapping SHFBG spectra for training their CNN-LSTM demodulation model (see Equation (11) in [15]). This confirms that the linear superposition model is considered a reasonable and practical approximation for algorithm validation in the FBG sensing community. We acknowledge that for serial FBG arrays where physical interactions between gratings are non-negligible, a more comprehensive model based on transfer-matrix formalism would be required; however, this is beyond the scope of the present work, which focuses on parallel WDM sensing networks.

#### 2.4.3. Discussion of Simulation-to-Reality Gap

It is important to acknowledge that the current evaluation was conducted on synthetically generated FBG spectra based on the asymmetric generalised Gaussian model [25], which has been experimentally validated to represent typical distortions induced by non-uniform strain and temperature gradients. However, practical FBG systems are subject to additional non-ideal effects that are difficult to fully capture in simulation, including mechanical vibrations, polarisation fluctuations, temperature drifts, input power variations, and connector-induced reflections. Some of these effects may have a non-negligible impact on demodulation performance. We therefore discuss the potential influence of these factors and the corresponding mitigation strategies inherent to our framework.

First, the multi-scale decomposition and bidirectional mixing mechanism of TimeMixer provides intrinsic robustness to certain types of non-ideal effects. Temperature-induced wavelength drift manifests as a slow baseline variation, which is naturally captured by the coarse-scale (trend) components and does not significantly affect the fine-scale peak features that determine the Bragg wavelength. Similarly, input power fluctuations and polarisation-dependent losses mainly affect the spectral amplitude rather than the peak position; the per-sample Z-score normalisation applied in our preprocessing effectively eliminates amplitude variations. These design choices provide a degree of intrinsic immunity to several common non-ideal factors.

Second, the modular architecture of our framework allows flexible integration of additional front-end preprocessing modules. For instance, the SAK-SVD denoising algorithm (presented in our previous work [26]) can be applied to suppress vibration-induced and other transient distortions before feature extraction, further enhancing robustness in demanding environments.

Nevertheless, we recognise that certain effects, such as polarisation-induced spectral deformation and nonlinear fibre distortions, are not explicitly modelled in the current simulation framework. To address this limitation, our ongoing work includes experimental validation on real FBG interrogators under controlled temperature and vibration conditions, as well as the collection of field-measured spectra from structural health monitoring installations. These future studies will quantitatively evaluate the generalisation capability of the proposed framework in practical engineering environments. The current simulation-based evaluation should therefore be viewed as an initial, yet necessary, step towards this goal, providing a controlled benchmark for algorithmic development.

### 2.5. Model Configuration and Hyperparameter Settings

The hyperparameters of all models were systematically optimised. The deep learning models employed the Adam or AdamW optimiser with an initial learning rate of 0.001 and weight decay of $1\times 10^{-5}$. Tree model parameters were determined via Bayesian optimisation. For double-peak tasks, the backbone structures remained unchanged, with only the output dimension adjusted to 2.

It should be emphasised that the hyperparameters of different models are inherently heterogeneous due to their distinct architectural designs—TCNs involve kernel sizes and channel depths, LSTM involves hidden units and recurrent layers, and LightGBM involves tree-specific parameters such as num_leaves and max_depth. Therefore, a fair comparison does not imply using identical hyperparameters across all models; rather, it requires that each model be independently optimised to its own best-performing configuration, so that the comparison reflects the true performance ceiling of each architecture rather than an arbitrary or suboptimal setting. To achieve this, we adopted a consistent optimisation methodology across all models: deep learning models (TCN, LSTM, GAF-CNN-LSTM, LSTM-CNN) were tuned through manual exploration guided by architectural best practices and grid search over key parameters; tree-based models (LightGBM, TCN-LightGBM) were optimised via Bayesian search to minimise validation error. Key hyperparameters are summarised in Table 1.

### 2.6. Evaluation Metrics

All experiments adopt unified evaluation metrics:

- Mean Absolute Error (MAE, nm): $\mathrm{MAE}=\frac{1}{N}\sum \left|{\hat{y}}_{i}-y_{i}\right|$.
- Root Mean Square Error (RMSE, nm): $\mathrm{RMSE}=\sqrt{\frac{1}{N}\sum {\left({\hat{y}}_{i}-y_{i}\right)}^{2}}$.
- Maximum Absolute Error (MAXE, nm): $\mathrm{MAXE}=\max|{\hat{y}}_{i}-y_{i}|$.

For double-peak tasks, the metrics were computed for each peak separately and are also reported as average MAE and overall RMSE.

Efficiency Metric: Single-sample inference time (ms), covering the full processing chain from input to output. The reported per-sample inference time was measured from the moment the pre-loaded spectral data (already residing in GPU memory) was fed into the model to the moment the final wavelength prediction was obtained. This measurement includes all GPU-resident operations: preprocessing (e.g., Z-score normalisation), TimeMixer forward propagation, LightGBM regression, and postprocessing (e.g., inverse standardisation). Data loading from disk and CPU-to-GPU data transfer were excluded from the timing, as all test data were pre-loaded into GPU memory prior to measurement. Measurements were performed on the NVIDIA RTX 4060 Ti GPU with CUDA 12.6, using a batch size of 64. Reported times are the average of 5 runs, with standard deviation <5%.

### 2.7. Selection of Comparison Models

Five representative models were selected for comparison:

1. Single deep learning models: TCN [17], LSTM [12].
2. Convolution-recurrent composite models: LSTM-CNN [13], GAF-CNN-LSTM [15].
3. Similar hybrid model: TCN-LightGBM [17].

### 2.8. Experimental Scheme Design

The following experiments were carried out sequentially:

1. Single-peak demodulation algorithm comparison.
2. Double-peak overlapping demodulation algorithm comparison (mild, moderate, severe overlap).
3. Ablation study (LightGBM alone, TimeMixer alone, TimeMixer-LightGBM).
4. Noise robustness analysis (SNR 5–40 dB).
5. Computational complexity and engineering deployment potential evaluation.

## 3. Results

### 3.1. Single-Peak Demodulation Performance Comparison

To comprehensively evaluate the fundamental demodulation capability of the proposed TimeMixer-LightGBM hybrid learning framework, a horizontal comparison was first conducted on the single-peak distorted spectrum dataset against five representative models: TCN, TCN-LightGBM, GAF-CNN-LSTM, LSTM, and LSTM-CNN. The experimental results are presented in Table 2.

Table 2 presents the comprehensive performance of the proposed TimeMixer-LightGBM model and five comparison methods on the single-peak distorted spectral dataset. To better understand the strengths and weaknesses of each approach, the following analysis addresses two perspectives: accuracy and speed.

In terms of accuracy, GAF-CNN-LSTM achieves a slightly better MAE (1.269 pm) and RMSE (2.117 pm) than the proposed method (1.656 pm and 2.128 pm), both reaching the same picometre level. However, on the MAXE metric, which reflects worst-case performance, the proposed method attains only 9.119 pm, a reduction of 61.3% compared with GAF-CNN-LSTM’s 23.560 pm. This significant difference can be attributed to three architectural features. First, the multi-scale decomposition of TimeMixer explicitly separates the coarse-scale baseline from the fine-scale peak details, ensuring that the critical peak morphology is preserved even under severe distortion. Second, the bidirectional mixing mechanism—bottom-up for seasonal components and top-down for trend components—prevents the amplification of localised distortions: fine-scale details are propagated upward to maintain feature fidelity, while clean baseline information is imposed downward to suppress noise-induced fluctuations. Third, LightGBM, as a gradient boosted tree ensemble, can more effectively suppress extreme errors through its threshold-based splitting and the voting mechanism of multiple trees. In contrast, although GAF-CNN-LSTM performs well on average, its linear output head is less robust to anomalous features, causing a few distorted samples to produce seriously deviated predictions. Consequently, the proposed method not only maintains high average accuracy but also provides more reliable worst-case guarantees.

Regarding speed, the proposed method achieves an inference time of 0.080 ms per sample, which is about 4.5 times faster than GAF-CNN-LSTM (0.363 ms). This total latency is decomposed into two components: TimeMixer feature extraction consumes 0.0767 ms (95.9% of the total), while LightGBM regression consumes only 0.0033 ms (4.1% of the total). This breakdown reveals that the regression stage adds negligible overhead, validating the efficiency of the decoupled “feature extraction + lightweight regression” design. This acceleration does not rely solely on GPU power but originates from two essential architectural features. First, TimeMixer adopts a pure MLP architecture whose core operations are matrix multiplications and fully connected layers. Unlike the sliding convolution windows of CNNs or the serial gated recurrences of LSTMs, matrix multiplications are highly parallelisable on hardware such as GPUs and FPGAs, with neither convolution kernel redundancy nor sequential dependencies along the time dimension. Second, LightGBM, as the back-end regressor, performs inference using only integer comparisons and simple conditional branches (decision tree paths), involving no floating-point matrix operations. Our measurements show that the LightGBM regression step consumes only 0.0033 ms, accounting for just 4.1% of the total inference time. This layered design of “lightweight front-end feature extraction + ultra-lightweight back-end regression” fundamentally eliminates computational bottlenecks, reducing the overall latency to the microsecond level.

To further contextualise the performance of the proposed method, we compared it with four conventional peak-detection approaches—direct peak detection, the centroid method, Gaussian fitting, and polynomial fitting—on the same single-peak dataset. The results are summarised in Table 3.

Gaussian fitting and polynomial fitting achieve Mean Absolute Errors of 4.1 pm, which are close to that of the proposed method (1.656 pm), but exhibit significantly larger Maximum Absolute Errors (98.1 pm vs. 9.119 pm), indicating that conventional fitting methods are prone to large deviations on severely distorted outlier samples. In contrast, the proposed method maintains consistent performance across all samples, with a MAXE improvement of approximately 10× over Gaussian fitting and a 40× speed advantage over Gaussian fitting (0.080 ms vs. 3.197 ms). This confirms that the proposed method not only achieves competitive average accuracy but also provides superior worst-case robustness and practical efficiency.

### 3.2. Performance Evaluation of Double-Peak Overlapping Demodulation

To investigate the potential of the proposed framework in dense wavelength-division multiplexing (WDM) sensing, experiments were conducted on three levels of double-peak overlapping datasets: mild, moderate, and severe. All comparison models were modified into a dual-output structure, with hyperparameters kept consistent with those used in the single-peak task, in order to evaluate the capability of each method in resolving overlapping peaks.

Table 4 presents the test results under mild-overlap conditions. Under mild overlap, GAF-CNN-LSTM achieves the best average MAE (0.0015 nm), followed closely by the proposed TimeMixer-LightGBM model (0.0031 nm) and TCN (0.0038 nm); all three are at the picometre level. However, LSTM and LSTM-CNN already exhibit average MAEs as high as 0.3578 nm, revealing the vulnerability of recurrent networks to overlapping spectra—even when the two peaks are still separated by a resolvable valley, their serial gating mechanisms cannot decouple two independent wavelength components.

Under moderate overlap, error divergence becomes evident. The average MAE of the proposed method increases only slightly from 0.0031 nm to 0.0036 nm (a 16% increase). GAF-CNN-LSTM rises from 0.0015 nm to 0.0017 nm (13% increase). In contrast, the MAE of TCN jumps from 0.0038 nm to 0.0152 nm—a 300% increase—indicating that its dilated convolution architecture struggles to maintain independent identification of the two peaks once the valley disappears. The MAEs of LSTM and LSTM-CNN drop to around 0.03 nm, but this is not a sign of successful decoupling; rather, because the two peaks have merged, the models can only guess a compromised wavelength, giving the illusion of reduced error while completely losing the ability to distinguish the two peaks.

Under severe overlap, the spectra almost merge into a single peak, and the two can only be distinguished by subtle asymmetries. The proposed method demonstrates a clear advantage: its MAE rises only to 0.0061 nm, representing a degradation factor of 1.97 from mild to severe overlap. GAF-CNN-LSTM reaches 0.0036 nm (factor 2.40), while TCN deteriorates to 0.0336 nm (factor 8.84), indicating that its deep dilated convolutions begin to suffer from severe inter-peak crosstalk under heavy aliasing, causing the two output channels to interfere with each other. LSTM and LSTM-CNN revert to MAEs around 0.44 nm, completely failing.

Table 5 summarises the average MAE variation across the three overlap levels. The much lower degradation rate of the proposed method stems from the intrinsic design of TimeMixer: its multi-scale bidirectional mixing mechanism explicitly decomposes the spectra into trend (baseline) and seasonal (detail) components, preserving subtle peak asymmetries even under extreme overlap.

Examining the error evolution from mild to severe overlap reveals distinctly different trends across models. For TCN, the MAE increases moderately from 0.0038 nm to 0.0152 nm (4.0×) between mild and moderate overlap, but then surges sharply to 0.0336 nm (2.2× from moderate to severe). This accelerated degradation indicates that once the valley between peaks disappears, TCN’s dilated convolutions lose the ability to distinguish individual peaks. In contrast, the proposed method exhibits a smooth and nearlinear error growth: MAE increases from 0.0031 nm to 0.0036 nm (1.16×) and then to 0.0061 nm (1.69×), with a consistent growth rate across both transition stages.

Inference time remains stable across all overlap levels (0.079 ms) because TimeMixer and LightGBM process each spectrum independently, regardless of peak relationships, incurring no additional computational overhead as spectral complexity increases.

### 3.3. Ablation Study

To verify the necessity of each component and the synergistic effect of the hybrid architecture, we designed three ablation groups: (1) standalone LightGBM, which directly regresses the centre wavelength from the raw spectrum without any feature extraction; (2) standalone TimeMixer, which uses the built-in linear projection head to map the extracted multi-scale features to the wavelength; (3) the complete TimeMixer-LightGBM hybrid framework. The standalone LightGBM group evaluates the feasibility of directly applying a tree-based regressor to the high-dimensional raw spectrum, i.e., the necessity of a dedicated feature extractor. The standalone TimeMixer group quantifies the regression capability of the pure MLP architecture alone, serving as a baseline for the contribution of the subsequent gradient boosting regressor. By comparing (3) with (1) and (2), we can quantitatively assess the synergistic gain of decoupling feature extraction (TimeMixer) from nonlinear regression (LightGBM)—a design that allows each module to focus on its respective strength. The experimental results are summarised in Table 6.

The test data show that the standalone LightGBM model suffers a severe degradation (MAE = 22.035 pm). The root cause lies in the fact that the core mechanism of tree models is based on numerical threshold splitting, which lacks the ability to perceive translation invariance in sequential signals. The wavelength shift of the spectrum manifests itself as a global translation on the numerical sequence, and relying solely on tree models makes it difficult to capture this spatial position mapping relationship from high-dimensional raw data. When the standalone TimeMixer network is adopted, the system performance leaps to an MAE of 2.195 pm, indicating that the network possesses a strong underlying feature interpretation capability and can map the raw spectral sequence into a deep feature space that encodes wavelength information. The final hybrid architecture further reduces the MAE by 24.6% (to 1.656 pm) and the MAXE by 32.1% compared with the standalone TimeMixer. This step improvement suggests that although TimeMixer is suitable for feature extraction, its built-in conventional linear regression head remains insufficient for fitting the highly nonlinear wavelength shift correlation. Introducing LightGBM as the back-end regression engine fully exploits the fitting advantages of ensemble learning when handling highly structured features, thereby achieving a pronounced algorithmic synergistic effect.

### 3.4. Noise Robustness Analysis

**Figure 7:**
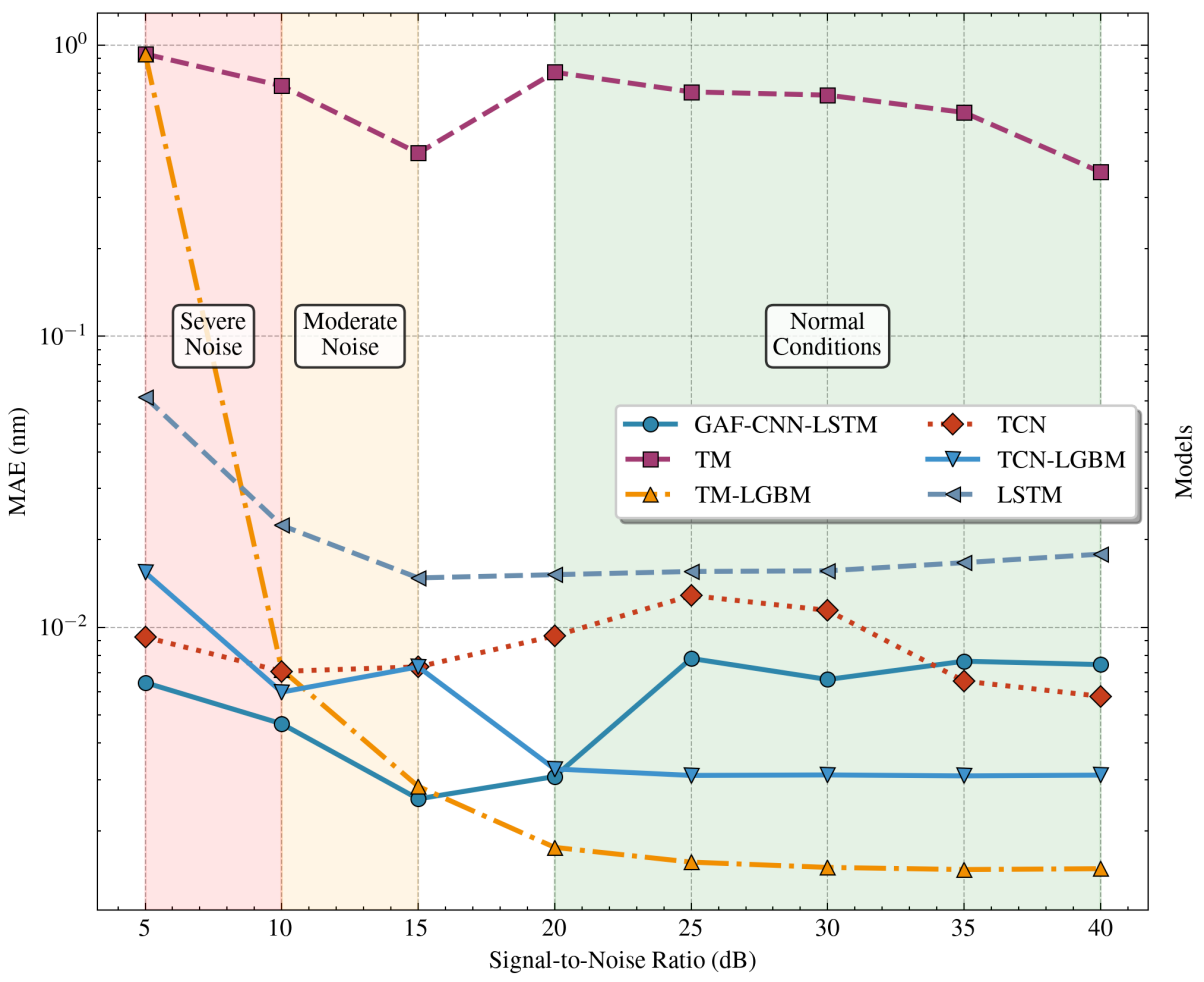
Noise experimental results: MAE vs. SNR.

In practical FBG sensing systems, the influence of light source fluctuations, electromagnetic interference, and environmental noise is unavoidable. To evaluate the stability of each algorithm in noisy environments, this study takes the single-peak dataset as the benchmark and tests each model over an SNR range of 5–40 dB. The results are presented in Figure 7.

Within the typical industrial SNR range of 20–40 dB, the proposed TimeMixer-LightGBM model maintains an exceptionally stable MAE of approximately 1.5 pm (specifically, 1.497–1.561 pm). Even under a low SNR of 15 dB, the MAE is only 2.842 pm, and at 10 dB it remains as low as 7.152 pm. By contrast, GAF-CNN-LSTM exhibits pronounced fluctuations: its MAE varies from 0.0026 nm (2.6 pm) at 15 dB to 0.0078 nm (7.8 pm) at 25 dB, with no clear monotonic trend and several peaks exceeding 0.007 nm. This non-monotonic behaviour—where MAE locally increases with improving SNR—is not due to data errors, but rather reflects the sensitivity of certain architectures to specific noise patterns; as the noise level changes, the spurious correlations learned by these models no longer hold, causing error rebound. This instability suggests that GAF-CNN-LSTM, relying on a fixed receptive field and a linear output head, is sensitive to specific noise realisations and lacks an explicit mechanism to separate signal from noise. The TCN series and LSTM-based models show even higher errors and greater variability.

The superior robustness of the proposed method originates from the multi-scale decomposition and bidirectional mixing mechanism of TimeMixer. The coarse-scale (trend) components capture the spectral envelope and baseline, which are inherently robust against high-frequency random noise, while the fine-scale (seasonal) components preserve peak details. The top-down trend mixing uses the clean baseline information to regularise the fine-scale estimates, effectively suppressing noise-induced fluctuations without blurring the peak shape. This gives TimeMixer a natural advantage over single-scale models. Furthermore, LightGBM, as a tree-based ensemble, is less sensitive to small input perturbations than neural network regressors, contributing additional robustness. As a result, the proposed framework not only achieves high precision but also maintains reliable and stable output across a wide SNR range (5–40 dB), with the MAE dropping sharply from 0.9321 nm at 5 dB to 0.0072 nm at 10 dB and then stabilising at the sub-picometre level, demonstrating excellent noise immunity and rapid recovery from extremely noisy conditions.

The above results indicate that the proposed framework possesses high precision, high real-time capability, and strong noise robustness, making it suitable for industrial environments where noise levels vary unpredictably. The noise robustness under double-peak overlapping conditions will be further verified in future work.

### 3.5. Computational Complexity Comparison

To quantitatively justify the architectural advantages of TimeMixer over existing deep learning models, we compare the computational complexity of all models involved in our experiments. Table 7 summarises the parameter count, floating-point operations (FLOPs), and per-sample inference time of each model. FLOPs were measured using the ptflops library with a single forward pass on the GPU, and inference time was measured on the test set (1500 samples) with a batch size of 64, including GPU-based preprocessing (Z-score normalisation) and postprocessing (inverse standardisation).

Table 7 reveals several key observations. First, TimeMixer achieves 12.15M parameters and 7218.46 MFLOPs, which is substantially lower than TCN (17.12 M, 136,042.02 MFLOPs) and GAF-CNN-LSTM (269.25 M, 651.54 MFLOPs). The FLOPs of TCN are approximately 18.8 times higher than those of TimeMixer, confirming that the pure MLP architecture of TimeMixer is significantly more computationally efficient than the dilated convolution stacks of TCN. Second, although GAF-CNN-LSTM has lower FLOPs (651.54 MFLOPs) than TimeMixer, its parameter count is 22 times larger (269.25 M vs. 12.15 M), leading to a model size of over 20 MB and higher memory bandwidth requirements. Third, LightGBM adds only 0.0033 ms to the inference time, accounting for just 4.1% of the total 0.08 ms, validating the “feature extraction + lightweight regression” decoupling design. Finally, the inference time of our hybrid framework (0.08 ms) is 4.5 times faster than GAF-CNN-LSTM (0.363 ms), directly translating the architectural efficiency into practical speed advantages. These quantitative results strongly support the hardware-friendliness and computational efficiency of the proposed TimeMixer-LightGBM framework.

The efficiency advantage of the proposed MLP+tree-based architecture can be further understood from three hardware-level perspectives. First, TimeMixer has no sequential dependency—its pure MLP operations are fully parallelisable matrix multiplications, unlike LSTM’s serial gating mechanism which processes time steps sequentially and prevents full parallelisation. Second, there is no convolution kernel redundancy—TimeMixer avoids the irregular memory access patterns of dilated convolutions in TCN, which require frequent off-chip memory accesses. Third, tree-based inference is exceptionally lightweight—LightGBM uses only integer comparisons and conditional branches, with no floating-point matrix operations, as reflected in the 0.0033 ms regression time. These factors collectively explain why the proposed architecture achieves both lower latency and better hardware compatibility.

## 4. Discussion

Based on the experimental results, the following core observations are made:

1. TimeMixer-LightGBM achieves the optimal balance between accuracy and speed in both single-peak and double-peak tasks. The single-peak MAE is 1.656 pm with an inference time of 0.08 ms; under severe double-peak overlap, the MAE remains 6.1 pm with an inference time of 0.07 ms, fully meeting the real-time high-precision demodulation requirements of large-scale FBG sensor networks.
2. The multi-scale mixing mechanism is key to addressing overlapping peak demodulation. As overlap increases, TCN’s accuracy deteriorates sharply and LSTM fails completely. TimeMixer, through hierarchical decomposition and mixing, decouples the two peaks even under severe overlap with a degradation factor of only 1.97, verifying the inherent adaptability of this architecture to aliased signals.
3. Feature quality determines the upper limit of the hybrid architecture. Both TCN-LightGBM and the proposed framework follow the “feature extraction + gradient boosting” paradigm, yet the former is inferior in both accuracy and robustness, indicating that the multi-scale features extracted by the MLP are more conducive to subsequent tree regression than dilated convolutional features.
4. Noise robustness and computational efficiency ensure prospects for engineering deployment. The proposed method exhibits stable performance over a wide SNR range of 15–40 dB, and the combination of a pure MLP structure with lightweight LightGBM is naturally compatible with embedded hardware such as FPGAs and DSPs.
5. Physical implementation and scalability: Beyond algorithmic validation, the proposed framework is designed with practical deployment in mind. Physical implementation pathway: The digitised spectra from a standard FBG interrogator (broadband source, circulator, photodetector, and ADC) can be processed by a heterogeneous digital platform. TimeMixer’s pure MLP architecture—consisting primarily of matrix multiplications—is highly amenable to FPGA acceleration due to its regular memory access patterns and minimal parameter count (0.07 million). This eliminates the irregular memory access overhead of dilated convolutions (TCNs) and the sequential dependency bottlenecks of recurrent units (LSTMs). LightGBM inference, involving only integer comparisons and conditional branches, can be executed on an embedded processor or mapped to FPGA using high-level synthesis tools. Previous work has demonstrated the feasibility of deploying neural network-based FBG demodulation on FPGA platforms [27,28], and the parameter efficiency of TimeMixer further reduces on-chip memory and DSP resource requirements. Scalability: From a computational perspective, the per-channel processing time remains essentially constant regardless of the number of sensors in a wavelength-division multiplexing (WDM) network, as both TimeMixer and LightGBM process each spectrum independently. From a deployment perspective, the modular design supports flexible configurations: (i) edge deployment on a single FPGA/SoC for low-power, real-time applications; (ii) a cloud/edge hybrid for large-scale networks where feature extraction is distributed across multiple edge nodes; and (iii) centralised processing on workstations for laboratory or infrastructure monitoring. These characteristics make the framework suitable for a wide range of practical FBG sensing applications, from small-scale setups to large-scale industrial monitoring networks.

## 5. Conclusions

This paper proposes a TimeMixer-LightGBM hybrid learning framework that decouples feature extraction from regression for FBG demodulation in complex environments. In the single-peak task, the framework achieves picometre-level accuracy (RMSE = 2.128 pm) at 0.08 ms per spectrum, approximately 4.5 times faster than GAF-CNN-LSTM. In double-peak overlapping tasks, the MAE increases from 3.1 pm to only 6.1 pm from mild to severe overlap (degradation factor 1.97× vs. 8.84× for TCN), confirming the decoupling capability of the multi-scale mixing mechanism. The hybrid architecture outperforms standalone LightGBM and TimeMixer, reducing MAE by 24.6% compared with TimeMixer alone. The framework also exhibits strong noise robustness (1.5 pm MAE over 20–40 dB SNR). The pure MLP architecture is inherently compatible with embedded hardware such as FPGAs, offering a foundation for portable high-real-time-performance FBG demodulators. Future work will focus on three directions: experimental validation with real FBG interrogators under varying temperature, strain, and vibration conditions to quantify the impact of non-ideal effects not covered in simulation; extension to multi-peak overlapping demodulation scenarios; and transplantation to embedded platforms such as FPGAs and DSPs. This real-data validation will be critical to bridging the gap between simulation-based algorithm development and practical engineering deployment.

## Figures and Tables

**Table 1:** Key hyperparameter configurations of each model.

Model	Hyperparameters	Optimisation Method
TimeMixer-LightGBM (proposed)	TimeMixer: d_model = 256, e_layers = 1, d_ff = 256, moving_avg = 25, lr = 0.001, batch_size = 64; LightGBM: Bayesian optimisation, weighted ensemble of three models with MAE/RMSE/Huber	TimeMixer: Manual tuning; LightGBM: Bayesian optimisation
GAF-CNN-LSTM [15]	CNN: 4 layers, filters = [6, 16, 32, 48]; LSTM: hidden_size = 512, layers = 2; GAF size: 56 × 56, dropout = 0.3	Manual tuning
LSTM [12]	hidden_size = 16, num_layers = 4, dropout = 0.3, batch_size = 16	Manual tuning
TCN [17]	num_channels = [64, 128, 256, 512, 1024, 1024, 512, 256, 128, 64]; kernel_size = 3, dropout = 0.25, batch_size = 64	Manual tuning
TCN-LightGBM [17]	TCN: Same as TCN model configuration; LightGBM: Bayesian optimisation, three-model ensemble	TCN: Manual tuning; LightGBM: Bayesian optimisation
LSTM-CNN [13]	LSTM: hidden_size = 128, num_layers = 3; CNN: out_channels = 5, kernel_size = 3, dropout = 0.2	Manual tuning

**Table 2:** Experimental results of algorithm comparison (single-peak).

Model	MAE (pm)	RMSE (pm)	MAXE (pm)	Inference Time (ms)
TimeMixer-LightGBM	1.656	2.128	9.119	0.0800
TCN	7.152	8.644	29.785	0.4484
TCN-LightGBM	3.139	4.777	28.613	0.4562
GAF-CNN-LSTM	1.269	2.117	23.560	0.3628
LSTM	15.790	19.979	61.401	0.0281
LSTM-CNN	13.832	17.349	70.801	0.0300

**Table 3:** Comparison with conventional peak-detection methods on the single-peak dataset.

Method	MAE (pm)	RMSE (pm)	MAXE (pm)	Time (ms)
Direct Peak	7.1	9.0	33.9	0.0026
Centroid	14.1	16.5	50.1	0.0162
Gaussian Fit	4.1	5.6	98.1	3.197
Polynomial Fit	4.1	5.5	97.8	0.0312
TimeMixer-LightGBM	1.656	2.128	9.119	0.080

**Table 4:** Performance comparison under mild-overlap conditions.

Model	P1 MAE	P2 MAE	Avg. MAE	RMSE	MAXE	Time (ms)
TimeMixer-LightGBM	0.0029	0.0033	0.0031	0.0043	0.0240	0.0790
GAF-CNN-LSTM	0.0015	0.0015	0.0015	0.0020	0.0134	0.488
LSTM	0.3589	0.3566	0.3578	0.4121	0.7755	0.026
TCN	0.0032	0.0045	0.0038	0.0049	0.0175	0.062
TCN-LightGBM	0.0065	0.0053	0.0059	0.0090	0.1202	0.117
LSTM-CNN	0.3590	0.3566	0.3578	0.4121	0.7710	0.008

**Table 5:** Comparison of average MAE (nm) under different overlap levels.

Model	Mild	Moderate	Severe	Growth Rate (Mild→Severe)
TimeMixer-LightGBM	0.0031	0.0036	0.0061	1.97×
GAF-CNN-LSTM	0.0015	0.0017	0.0036	2.40×
LSTM	0.3578	0.0279	0.4379	1×
TCN	0.0038	0.0152	0.0336	8.84×
TCN-LightGBM	0.0059	0.0084	0.0102	1.73×
LSTM-CNN	0.3578	0.4023	0.4379	1×

**Table 6:** Ablation experiment results.

Model	MAE (pm)	RMSE (pm)	MAXE (pm)	Inference Time (ms)
LightGBM	22.035	59.117	768.573	0.007
TimeMixer	2.195	2.920	13.428	0.0767
TimeMixer-LightGBM	1.656	2.128	9.119	0.0800

**Table 7:** Computational complexity comparison of representative architectures.

Model	Params (M)	FLOPs (MFLOPs)	Inference Time (ms)
TimeMixer (this work)	12.15	7218.46	0.0800
LightGBM (this work)	3.26	—	0.0033
TCN	17.12	136,042.02	∼0.36
LSTM	0.01	33.55	0.0281
LSTM-CNN	0.33	1348.85	0.0300
GAF-CNN-LSTM	269.25	651.54	0.3628

## Data Availability

The synthetic data used in this study were generated by the analytical model described in Section 2.3.1 (Equation (12)) and Section 2.4. The data generation code is available on request from the authors. The datasets presented in this study are available within the article.

## Abbreviations

FBG	Fibre Bragg Grating
MLP	Multi-Layer Perceptron
PDM	Past-Decomposable-Mixing
FMM	Future-Multipredictor-Mixing
LightGBM	Light Gradient Boosting Machine
TCN	Temporal Convolutional Network
LSTM	Long Short-Term Memory
CNN	Convolutional Neural Network
SNR	Signal-to-Noise Ratio
MAE	Mean Absolute Error
RMSE	Root Mean Square Error
MAXE	Maximum Absolute Error
WDM	Wavelength-Division Multiplexing
